# Insulin-like growth factor 1 and 2 (IGF1, IGF2) expression in human microglia: differential regulation by inflammatory mediators

**DOI:** 10.1186/1742-2094-10-37

**Published:** 2013-03-12

**Authors:** Hyeon-Sook Suh, Meng-Liang Zhao, Leandra Derico, Namjong Choi, Sunhee C Lee

**Affiliations:** 1 Department of Pathology, Albert Einstein College of Medicine, Bronx, NY, 10461, USA

**Keywords:** Brain, Cytokines, Growth factors, HIV, Human, IGF1, IGF2, Inflammation, LPS, Microglia

## Abstract

**Background:**

Recent studies in experimental animals show that insulin-like growth factor 1 (IGF1) plays a trophic role during development and tissue injury and that microglia are important sources of IGF1. However, little information is available regarding the expression, regulation, and function of IGF1 and related proteins in human brain cells. In the current study, we examined the expression of IGF1 and IGF2 in human microglia *in vivo* and *in vitro*.

**Methods:**

Expression of IGF1 and IGF2 was examined by immunohistochemistry in post-mortem human brain sections derived from HIV+ and HIV− brains. In primary cultures of human fetal microglia, IGF1 and IGF2 mRNA and protein expression was examined by Q-PCR, ELISA, and Western blot analysis. Additionally, the role of IGF1 and IGF2 in neuroprotection was examined in primary human neuronal glial cultures.

**Results:**

Immunohistochemistry of human brain tissues showed that nonparenchymal cells (vessels and meninges), as well as parenchymal microglia and macrophages were positive for IGF1, in both HIV encephalitis and control brains, while IGF2 was undetectable. Cultured microglia expressed IGF1 mRNA and produced pg/ml levels of IGF1 protein; this was significantly suppressed by proinflammatory mediators, such as lipopolysaccharide (LPS), poly(I:C), and IFNγ. The Th2 cytokines IL-4 and IL-13 had no significant effect, but the cAMP analog (dibutyryl cAMP) significantly increased IGF1 production. In contrast, microglial IGF2 mRNA and protein (determined by Western blot) were upregulated by LPS. IGF1 receptor (IGF1R) immunoreactivity was predominantly expressed by neurons, and both IGF1 and IGF2 significantly protected neurons from cytokine (IL-1/IFNγ) induced death.

**Conclusions:**

Our study in human brain tissues and cells indicates that microglia are important sources of neurotrophic growth factors IGF1 and IGF2, and that microglial activation phenotypes can influence the growth factor expression. Importantly, our results suggest that chronic neuroinflammation and upregulation of proinflammatory cytokines could lead to neurodegeneration by suppressing the production of microglia-derived neuronal growth factors, such as IGF1.

## Background

Recent advances in microglial biology have revealed that microglia may have important homeostatic functions [1]. Microglia in normal brain are very active in surveillance of the normal neuronal environment and are the first cells to respond to any subtle changes [2]. Furthermore, not all activated microglia are toxic: microglia can contribute to neural repair and regeneration through phagocytosis and the production of anti-inflammatory and immunoregulatory mediators, as well as neuronal growth factors [3-6]. Recent studies in mice also indicate that microglia are distinct from monocytes in their origin, and that the two myeloid populations may have somewhat separate antigenic and functional profiles [7,8]. These findings together indicate that elucidating the microglial activation phenotypes and their regulatory mechanisms is important in understanding of the pathogenesis of diseases of the central nervous system (CNS).

In mice, the macrophage and microglial activation phenotypes are defined by the arginine metabolism pathway, with the classically activated phenotype (M1) characterized by the expression of inducible nitric oxide synthase (iNOS) and the alternatively activated phenotype (M2) by the expression of arginase-1 [9,10]. Macrophage M1 and M2 activation can typically be induced by exposure to Th1 (IFNγ) and Th2 (IL-4 or IL-13) cytokines, respectively. The primary role of M2 macrophages is in wound healing and tissue repair, with arginase-1 contributing to cell proliferation, resolution of inflammation, and remodeling of the extracellular matrix [9]. Considerably less information is available on the role of M2 microglia, but there is evidence that microglia produce neuronal growth factors IGF1, IGF2, and brain-derived growth factor (BDNF) [6,11-13]. To what extent the M1/M2 paradigm applies to human macrophages and microglia is uncertain, since neither iNOS nor arginase 1 is expressed in these cells [14,15].

IGF1 has emerged as a crucial factor in the CNS; it is involved in normal cognitive function and successful aging, in addition to development [16-19]. IGF1 belongs to the insulin-like growth factor (IGF) family of proteins, to which the agonists IGF1 and IGF2, the receptors IGF1R and IGF2R, and several IGF-binding proteins (IGFBP1-7) belong [20-22]. Both IGF1 and IGF2 signal through the IGF1R leading to the growth and metabolic effects via the downstream PI3K/Akt pathway. IGF2R is not a signaling receptor but is instead involved in the capture and degradation of extracellular IGF2 (and IGF1) during development [23]. Although circulating blood IGF1 and IGF2 levels exert trophic effects on neurogenesis and neuronal survival [18,20,24], CNS-derived or intrathecally derived IGF1 is also important in maintaining normal brain function [25]. It has been shown that local and systemic levels of IGF1 are altered in such CNS diseases as Alzheimer’s disease [26]. IGF1, IGF2, IGFBP2, and IGFBP3 levels are also altered in individuals with HIV infection [27,28]. We have also recently reported that IGF2R is highly upregulated in HIV-infected CNS and that IGF2R is a novel IFNγ-inducible microglial protein that functions as a positive regulator of HIV infection [29]. Given these provocative findings and the significant species-dependent differences in neuroinflammatory mechanisms, we examined the regulation of IGF1 and IGF2 expression in human microglia *in vivo* and *in vitro*.

## Methods

### Human brain sections and IGF1 and IGF2 immunohistochemistry

Post-mortem brain sections with neuropathologic diagnosis of HIV encephalitis (HIVE) or minimal nonspecific changes (normal-appearing brains from both HIV+ and HIV− individuals) were obtained from the Manhattan HIV Brain Bank and processed for immunohistochemistry as previously described [29-31]. Briefly, sections were deparaffinized and underwent antigen retrieval in a citrate buffer at 95°C for 20 min. The primary antibody used was a rabbit polyclonal antibody (prediluted) against full-length recombinant human IGF1 (Abcam, catalog no. ab15320) based on its reaction on control sections. Positive controls for IGF1 included paraffin-embedded human fetal brain (strong staining of meninges and meningeal macrophages), as well as human fetal liver and the term placenta. Negative controls were (1) sections incubated with irrelevant antibodies and (2) sections incubated with antibodies pre-absorbed with the IGF1 peptide (Abcam). Sections were incubated overnight with the prediluted antibody at 4°C and then with anti-rabbit micropolymer-linked secondary antibody (ImmPress kit, Vector Laboratories) following the manufacturer’s instructions. Color was developed using diaminobenzidine. IGF2 immunohistochemistry was performed using a rabbit antibody from Abcam (ab9574) that had previously been used to characterize human tumor specimens [32], employing the approaches described for IGF1. Briefly, the sections were incubated overnight with the IGF2 antibody (dilution of 1:100) at 4°C and then with anti-rabbit micropolymer-linked secondary antibody (ImmPress kit) following the manufacturer’s instructions.

### IGF1R immunocytochemistry

The expression of IGF1R in mixed human CNS cell culture was determined by immunohistochemistry using anti-IGF1R antibody from R&D systems (MAB391), as previously described [33]. Mixed human CNS cell cultures were fixed in 100% ice-cold methanol and were then permeabilized with 0.1% Triton X-100 detergent in PBS. Blocking for endogenous peroxidase and for nonspecific binding was performed with 3% H_2_O_2_, followed by 5% normal goat serum (NGS) in PBS, each for 30 min. Cultures were incubated with primary antibody (dilution of 1:1000) overnight at 4°C and then with the mouse micropolymer-linked secondary antibody (ImmPress kit) following the manufacturer’s instructions. Color was developed using diaminobenzidine.

### Microglial culture

Human CNS cell cultures were prepared from human fetal abortuses (gestational ages from 16 to 20 weeks) as previously described [34] with minor modifications. All tissue collection was approved by the Albert Einstein College of Medicine Institutional Review Board. Primary mixed CNS cultures were prepared by enzymatic and mechanical dissociation of the cerebral tissue followed by filtration through nylon meshes of 230- μm and 130-μm pore size. Single cell suspensions were plated at 1 to 10 × 10^6^ cells per ml in DMEM (Cellgro, now ThermoFisher Scientific) supplemented with 10% FBS (Gemini Bio-products, Woodland, CA), penicillin (100 U/ml), streptomycin (100 μg/ml) and Fungizone (0.25 μg/ml) (complete medium) for 2 weeks, and then microglial cells were collected by aspiration of the culture medium. Monolayers of microglia were prepared in 60-mm tissue culture dishes at 1 × 10^6^ cells per 5 ml medium (for Q-PCR) or in 96-well tissue culture plates at 4 × 10^4^ per 0.1 ml medium (for ELISA). Four to eighteen hours later, cultures were washed to remove nonadherent cells (neurons and astrocytes). Microglial cultures were highly pure, and consisted of >98% CD68^+^ cells.

### Culture stimulation

The culture medium was changed to low-serum medium (DMEM plus 0.05% FBS) before cell stimulation to reduce potential effects from other growth factors. Lipopolysaccharide (LPS) and poly(I:C) were purchased from Sigma-Aldrich (St. Louis, MO) and used at 100 ng/ml and 10 μg/ml, respectively. Recombinant human cytokines (IL-1β, IFNγ, IL-4, and IL-13) were purchased from Peprotech (Rocky Hill, NJ) and were used at 10 ng/ml, unless otherwise stated. Microglia were also stimulated with dibutyryl adenosine 3′, 5′-cyclic monophosphate (db cAMP, Sigma-Aldrich) at 0.5 to 5 mM, or with recombinant growth hormone (R&D Systems, catalog no. 1067-GH) at 1 to 100 ng/ml. Cells were stimulated and harvested as specified in individual experiments.

### Q-PCR

Quantitative real-time reverse-transcription PCR (Q-PCR) was performed as described [35,36], using porphobilinogen deaminase (PBDA) as an internal control. Briefly, total RNA was extracted with TRIzol and PCR performed using a SYBR green PCR mix and the ABI Prism 7900HT system (Applied Biosystems). All values were expressed as the increase relative to the expression of PBDA. The median value of the replicates for each sample was calculated and expressed as the cycle threshold (C_T_; cycle number at which each PCR reaches a predetermined fluorescence threshold, set within the linear range of all reactions): ΔC_T_ was calculated as C_T_ of endogenous control gene (PBDA) minus C_T_ of target gene in each sample. The relative amount of target gene expression in each sample was then calculated as 2^ΔCT^. Fold change was calculated by dividing the value (2^ΔCT^) of test sample by the value (2^ΔCT^) of control sample (control = 1). Primers for Q-PCR were IGF1: forward TGGATGCTCTTCAGTTCGTG, backward TGGTAGATGGGGGCTGATAC; IGF2: forward ACACCCTCCAGTTCGTCTGT, backward GGGGTATCTTGGGGAAGTTGT; IGF2R: forward GAAGGTGAAGGTCGGAGT, backward GAAGATGGTGATGGGATTTC; TNFα: forward GGCGTGGAGCTGAGAGATAAC backward, GGTGTGGGTGAGGAGCACAT.

### ELISA

The levels of IGF1 and IGF2 were determined using human IGF1 antibody DuoSet (catalog no. DY291, detection limit ~50 pg/ml) purchased from R&D Systems (Minneapolis, MN) and IGF2 ELISA kit (catalog no. RHF350CK, detection limit ~300 pg/ml) from Antigenix America (Huntington Station, NY) respectively, following the manufacturer’s instructions. Briefly, polystyrene 96-well plates (Nunc) were pre-coated overnight at room temperature with specific capture Ab, then blocked with 1% BSA in buffer A (PBS plus 0.1% Tween 20) for 2 h at room temperature. The plates were then incubated with standard IGF dilutions or cell culture media for 2 h at room temperature, washed with buffer A, and incubated with the biotinylated detection Ab for 2 h at room temperature. After the second wash, the plates were incubated with HRP-streptavidin for 30 min at room temperature and washed again. The signal was developed after addition of 3,3',5,5'-tetramethylbenzidine-peroxidase EIA kit (Bio-Rad) for 4 to 10 min and the reaction was stopped by 1M H_2_SO_4_. A microplate reader (Dynex Technologies) was used to detect the signals at 450 nm with correction at 530 nm.

### Western blot

Western blot analysis was performed as previously described [11,35] with minor modifications. Briefly, cell cultures in 60 mm dishes were scraped into lysis buffer (PBS plus protease inhibitors from Sigma) at various time points. Thirty micrograms of protein was separated by 4 to 20% TGX polyacrylamide gel electrophoresis and then transferred to a polyvinylidene difluoride membrane. The blots were blocked in PBS-0.1% Tween-20 containing 5% nonfat milk and then incubated with antibodies at 4°C for 16 h. Primary antibodies were against rabbit polyclonal IGF2 from Abcam (ab9574), applied at a dilution of 1:500. An antibody against β-actin (Sigma-Aldrich, A2228) or vinculin (Santa Cruz, SC5573) was used as the loading control. The secondary antibody was either horseradish peroxidase-conjugated anti-mouse or anti-rabbit IgG (Pierce Biotechnology, Rockford, IL) and was used at 1:1,000 for 1 h at room temperature. Signals were developed using enhanced chemiluminescence (Pierce Biotechnology). Densitometric analysis was performed using Scion NIH Image software (Scion, Frederick, MD).

### Preparation of mixed CNS cell cultures and neurotoxicity assay

Mixed neuronal and glial cultures were generated by replating the initial CNS cell cultures once into 96-well tissue culture plates, as previously described [34,37]. Cultures were kept as monolayers in DMEM with 10% FBS and antibiotics and used at approximately 4 to 6 weeks *in vitro*. Cultures were stimulated with IL-1/IFNγ at 10 ng/ml in low-serum medium (DMEM + 0.05% FBS) for 72 h to induce neurotoxicity with or without recombinant human IGF1 (catalog no. 100–11) and IGF2 (catalog no. 100–12) purchased from Peprotech (NJ, Rocky Hill) at 10 ng/ml. Neurotoxicity assay was performed by vital dye exclusion (trypan blue) and microtubule-associated protein 2 (MAP2) immunostain, as previously described [37-39].

### Statistical analysis for tissue culture studies

Normalized data (fold change over control) from different brain cases were compared using one sample *t* test. For multiple comparisons, one-way analysis of variance (ANOVA) with Dunnett’s multiple comparison tests was performed. All statistics were performed using the GraphPad Prism 5.0 software.

## Results

### IGF1 is expressed in microglia and macrophages in human CNS

**Figure 1:**
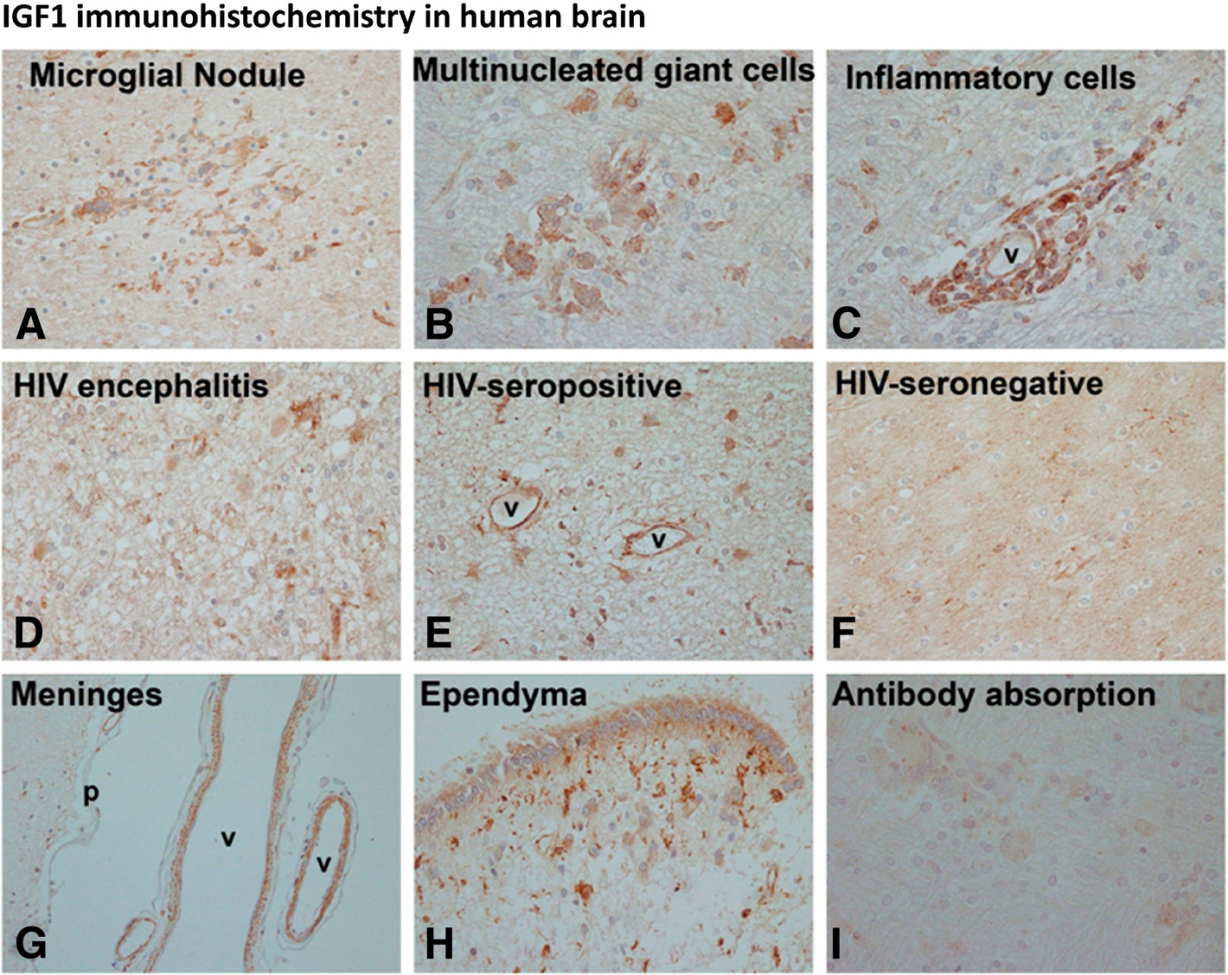
**IGF1 is expressed in human brain.** IGF1 immunohistochemistry was performed on paraffin-embedded post-mortem human brain sections from HIV+ and HIV− individuals. IGF1 staining was found in microglial nodules and multinucleated giant cells as well as perivascular inflammatory cells (perivascular lymphocytes and macrophages) in HIV encephalitis brains **(A-C)**. Examples of diffuse parenchymal microglial cell staining are shown in HIV encephalitis, HIV+ and HIV− brains **(D-F)**. Additional IGF1+ cell types included occasional endothelial cells (E: v = vessels) and astrocytes (in E). IGF1 staining was also noted in the meninges **(G)** in vascular smooth muscle cells (v = vessels), as well as in ependymal cells **(H)** in some brains (note strong microglial staining in the subependymal area in H). Controls included incubation of sections with the peptide pre-absorbed antibody, which abrogated IGF1 immunoreactivity **(I)**.

Results of IGF1 immunohistochemistry in human CNS using the HIV+ and HIV− brains are summarized in Figure 1 and in Table 1. In all of these brains, the main IGF1 immunoreactivity in the brain parenchyma was detected in myeloid cells (microglia and macrophages). For example, microglial nodules and multinucleated giant cells (MGCs) (from HIV-infected microglia and macrophages), the hallmark lesions of HIVE, were positive for IGF1 (Figure 1A,B). In addition, perivascular macrophages and inflammatory cells were also positive (Figure 1C). Parenchymal ramified microglial cells were IGF1 positive in all three conditions (HIVE, HIV+, and HIV− brains) (Figure 1D-F). In addition, occasional endothelial cells and vascular smooth muscle cells within the brain (Figure 1E) and meninges (Figure 1G), as well as ependymal cells (Figure 1H) and rare astrocytes (Figure 1E) or neurons (not shown) were IGF1-positive. A semiquantitative assessment of IGF1 immunoreactivity is presented in Table 1. These results together suggest that cells of myeloid lineage (microglia and macrophages) are the predominant IGF1-expressing cells in human CNS parenchyma, but other cells (including meningeal and vessel-associated cells) also contribute to the IGF1 production.

**Table 1:** IGF1 expression in human brain

**Pathology**	**Case number**	**Microglia**	**Multinucleated giant cells**	**Microglial nodules**	**Inflammatory cells**^**a**^	**Vessels and meninges**^**b**^	**Other**^**c**^
**HIV encephalitis**^**d**^	**Case 23**	**+**	**+**	**+**	**±**	**+**	**ependyma**
	**Case 1**	**+**	**+**	**−**	**+**	**+**	**ependyma**
	**Case 3**	**±**	**+**	**−**	**+**	**+**	**−**
	**Case 20**	**+**	**+**	**+**	**+**	**+**	**ependyma, neurons**
	**Case 22**	**+**	**+**	**+**	**+**	**+**	**−**
	**Case 9**	**+**	**+**	**+**	**±**	**+**	**−**
**No encephalitis**^**e**^	**Case 11 (HIV+)**	**+**	**NA**^**f**^	**NA**^**f**^	**NA**^**f**^	**+**	**−**
	**Case 12 (HIV+)**	**+**	**NA**^**f**^	**NA**^**f**^	**NA**^**f**^	**+**	**−**
	**Case 21(HIV+)**	**+**	**NA**^**f**^	**NA**^**f**^	**+**	**+**	**astrocytes**
	**Case 18 (HIV−)**	**+**	**NA**^**f**^	**NA**^**f**^	**NA**^**f**^	**+**	**neurons**

^a^Inflammatory cells were mononuclear cells (lymphocytes, monocytes and macrophages) present in the brain parenchyma or in perivascular spaces; ^b^IGF1 immunoreactivity was regularly associated with vessels in both brain parenchyma and meninges. Staining was present in smooth muscle cells, as well as in some endothelial cells. Some meningeal arachnoid cells and macrophages were also occasionally IGF1 positive; ^c^Other parenchymal IGF1positive cells included ependymal cells, occasional astrocytes or rarely even neurons; ^d^HIV encephalitis sections had characteristic pathology on H&E consisting of MGCs, microglial nodules, activated microglia and macrophages, and mononuclear inflammatory cell infiltration; ^e^Control brain sections had no significant pathology on H&E;

^f^NA (not applicable, since no inflammatory cells were present).

We also attempted to determine IGF2 expression in human CNS by immunohistochemistry. A rabbit polyclonal antibody from Abcam (ab9574), which had previously been used to characterize human tumor specimens [32], was utilized in this study. Using the approach described for IGF1 immunohistochemistry, we examined the same control and HIV+ brain sections. All brain sections showed diffuse background-like staining combined with some nuclear staining (data not shown). The nuclear staining was also observed in cultured microglia (not shown). Attempts with additional commercial IGF2 antibodies were equally unsuccessful, indicating either that the antibodies are not suitable for tissue immunohistochemistry or that there is no significant IGF2 expression in human CNS (see Discussion).

### Regulation of cultured human microglial IGF1 and IGF2 mRNA expression by inflammatory mediators (Q-PCR)

**Figure 2:**
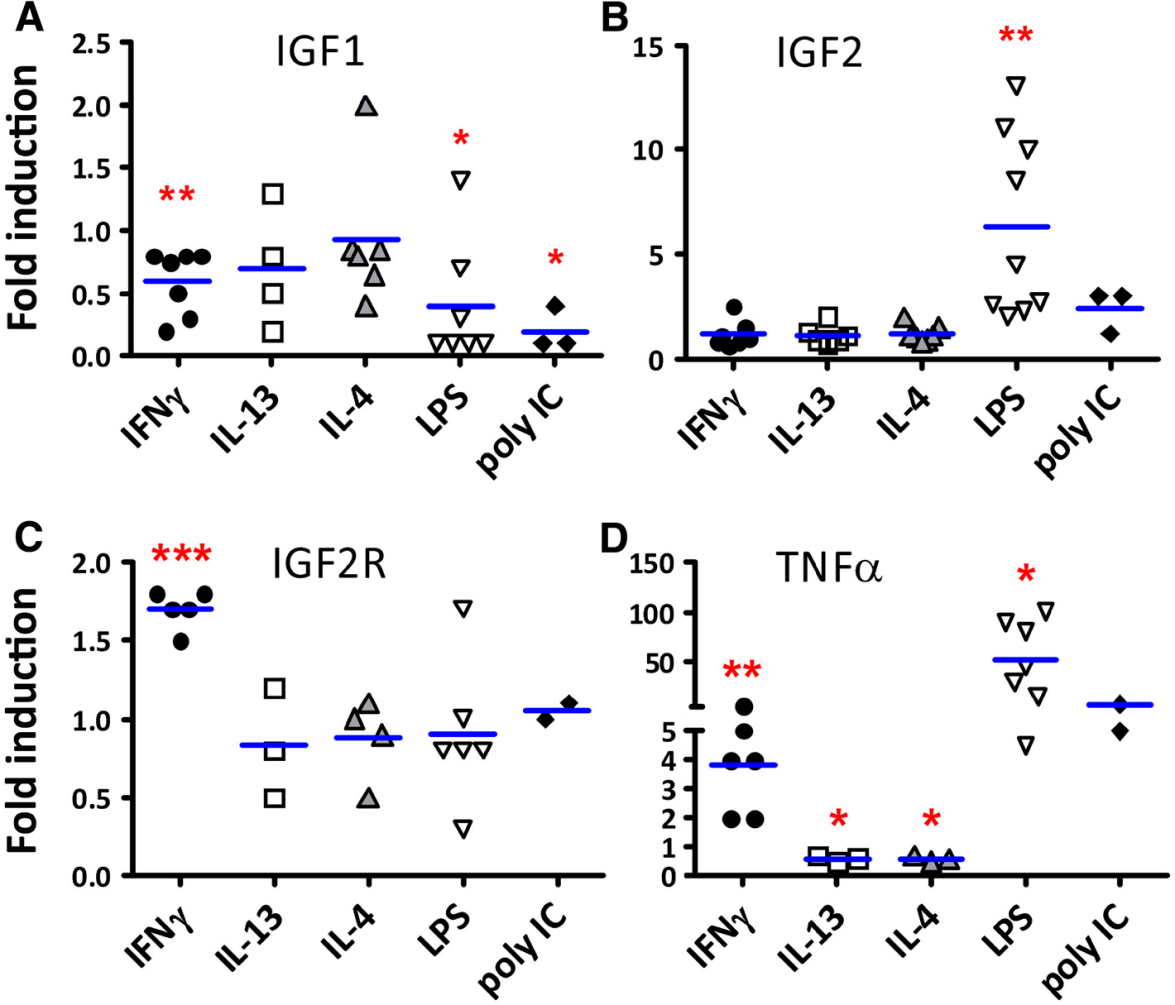
**Assessment of human microglial IGF1 and IGF2 mRNA expression by Q-PCR.** Microglia cultures were treated with IFNγ (10 ng/ml), IL-13 (10 ng/ml), IL-4 (10 ng/ml), LPS (100 ng/ml), poly(I:C) (10 μg/ml), or medium alone for 6 h and Q-PCR was performed for IGF1, IGF2, IGF2R, and TNFα. Fold changes over control (medium alone) were calculated in each case and values from multiple microglial cases were pooled and statistical significance analyzed using one sample *t* test (GraphPad Prism 5) * *P* < 0.05, ** *P* < 0.01, *** *P* < 0.001. IGF1 mRNA was suppressed by IFNγ, LPS, or poly(I:C) **(A)**, IGF2 mRNA increased by LPS **(B)**, IGF2R mRNA increased by IFNγ **(C)**, and TNFα mRNA increased by IFNγ, LPS or poly(I:C) (see broken *y* axis) and suppressed by IL-4 or IL-13 **(D)**.

We next examined the expression of IGF1 and IGF2 in primary human microglial cultures. The expression of IGF2R, which we previously reported to be an IFNγ-inducible novel microglial protein [29], and the proinflammatory cytokine TNFα were also examined simultaneously in the same cultures. Microglia were stimulated with Th1 (IFNγ) or Th2 (IL-4, IL-13) cytokines, the toll-like receptor (TLR) ligands (LPS or poly(I:C)), or medium alone (DMEM + 0.05% FBS) for 6 h at indicated doses, then mRNA expression was determined by Q-PCR. The mRNA levels for IGF1, IGF2, IGF2R, and TNFα were expressed relative to those in unstimulated cultures (control = 1). Results pooled from several microglial cultures derived from different brain cases are shown in Figure 2. They indicate that IGF1 mRNA expression was slightly suppressed by IFNγ but was more potently suppressed by LPS and poly(I:C) (Figure 2A). The effects of IL-4 and IL-13 were variable, with no significant effects across different cases. In contrast, IGF2 mRNA was significantly upregulated by LPS (Figure 2B), while IFNγ, IL-4, IL-13, and poly(I:C) had no significant effect. IGF2R mRNA was upregulated by IFNγ only (Figure 2C), consistent with our previous results obtained with Western blot [29]. TNFα mRNA was determined to control for the efficacy of cytokine and TLR ligands, and this showed that LPS and poly(I:C) potently induced TNFα mRNA, as expected. In addition, a small but significant induction and reduction of TNFα mRNA was also observed by IFNγ and IL-4/IL-13, respectively (Figure 2D, see Discussion). These results together show that the expression of IGF1, IGF2, IGF2R, and TNFα is distinctly regulated in human microglia.

### Regulation of human astrocyte IGF1 and IGF2 mRNA expression by inflammatory mediators (Q-PCR)

**Figure 3:**
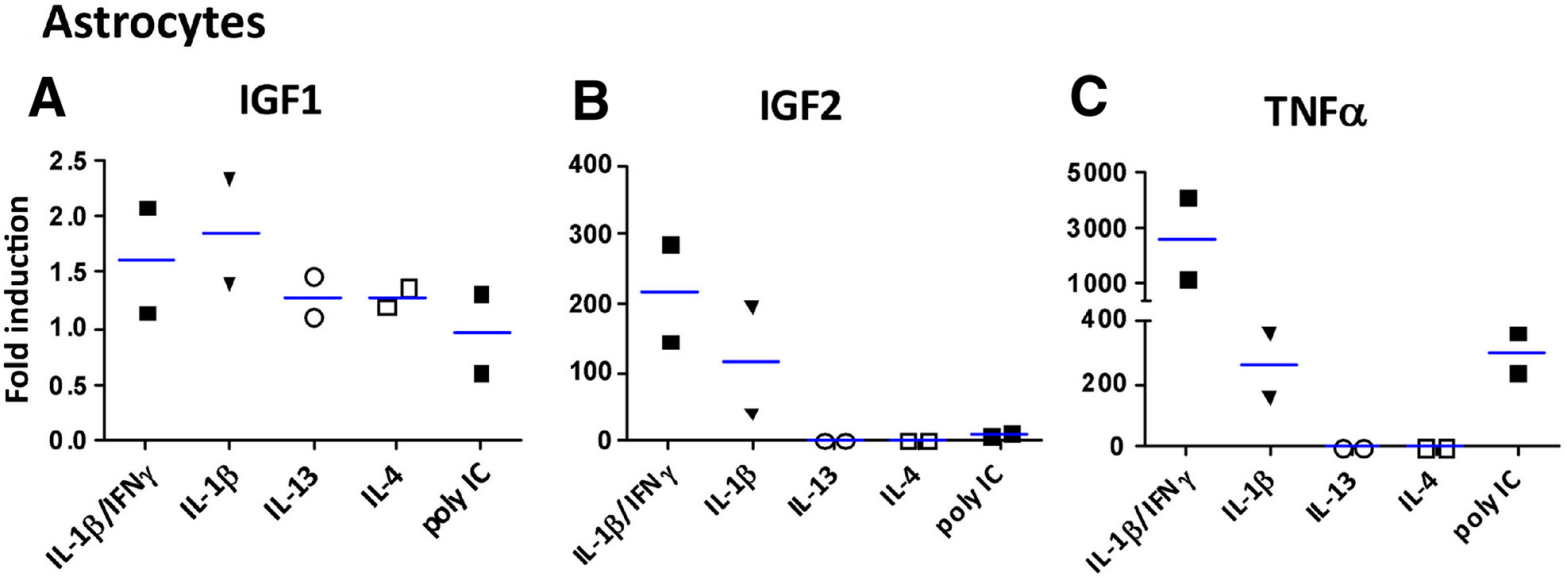
**Assessment of human astrocyte IGF1 and IGF2 mRNA expression by Q-PCR.** Astrocyte cultures were treated with IL-1β (±IFNγ) IL-13, IL-4, poly(I:C) or medium alone for 6 h and Q-PCR was performed for IGF1, IGF2, and TNFα. Fold changes over control were calculated in each case and values from two astrocyte cases are shown (bar = mean value). No significant changes were noted in astrocyte IGF1 mRNA following treatment with inflammatory stimuli **(A)** but IGF2 mRNA was potently induced by IL-1β (±IFNγ) **(B)**. TNFα mRNA was induced maximally by IL-1β/IFNγ and less by IL-1β or poly(I:C) but not by IL-13 or IL-4 **(C)**.

We have also performed Q-PCR analysis of astrocyte cultures for comparison with microglia. Since human astrocytes do not respond to LPS but respond maximally to IL-1, we treated cultures with the Th1 cytokine (IFNγ) with and without IL-1β, the Th2 cytokines (IL-4, IL-13), and the TLR ligand poly(I:C). The mRNA levels for IGF1, IGF2, and TNFα were determined and expressed relative to those in unstimulated cultures. Results from two different astrocyte cases are shown in Figure 3. Astrocyte IGF1 mRNA levels were not significantly changed by inflammatory mediators, while IGF2 mRNA levels were highly upregulated by IL-1β (±IFNγ). TNFα was also highly induced by IL-1β/IFNγ, as previously reported. Both IL-1β and poly(I:C) had lesser effects, while IL-4 and IL-13 had no effect. IGF1 was undetectable in astrocyte culture supernatants (not shown). These results suggest that while astrocyte IGF1 expression might be insignificant, IGF2 might be upregulated under inflammatory conditions (see Discussion).

### Regulation of human microglial IGF1 protein expression (ELISA)

**Figure 4:**
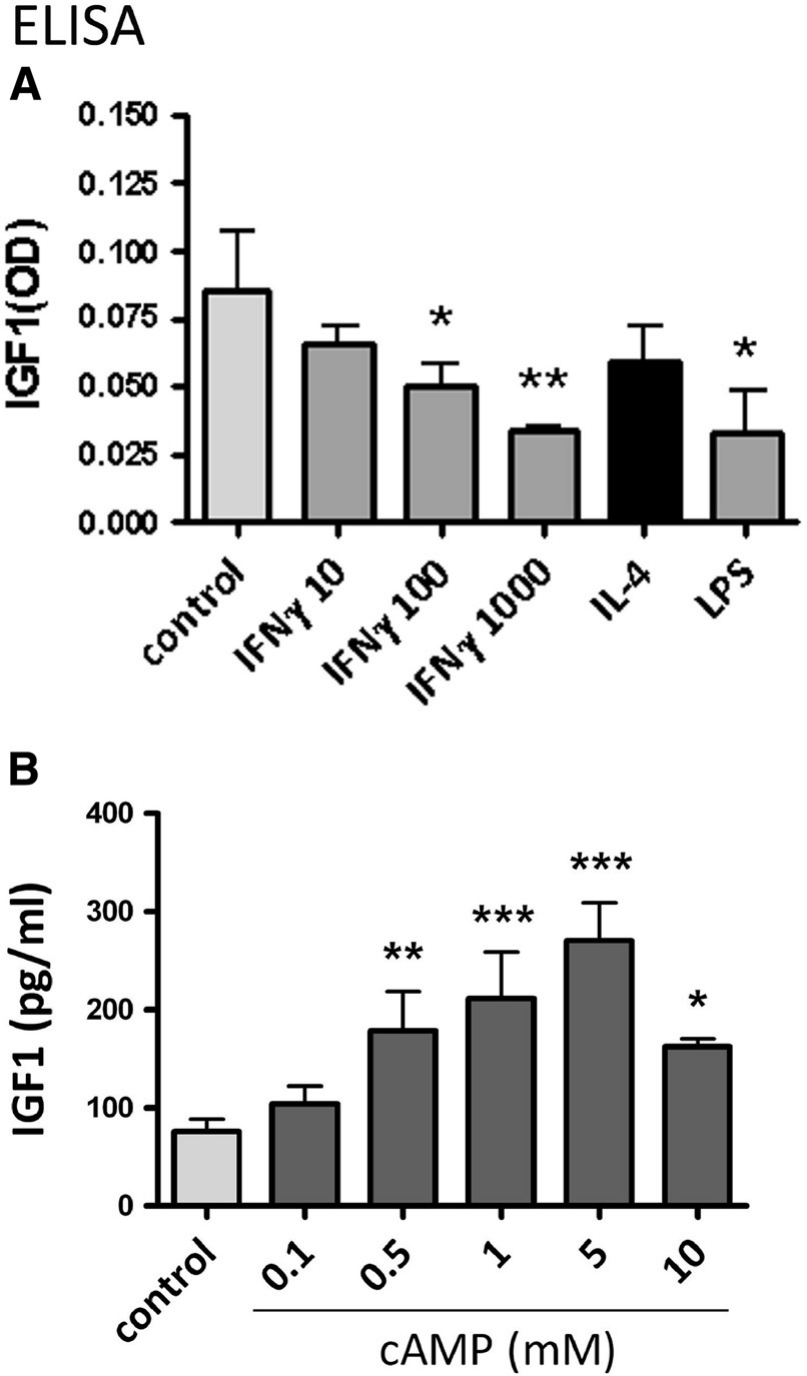
**IGF1 protein production by microglia as assessed by ELISA.** Microglia in triplicate cultures were stimulated with IFNγ (10, 100, and 1000 ng/ml), IL-4 (10 ng/ml), LPS (100 ng/ml), or medium alone (control) for 24 h and culture supernatants were analyzed for IGF1 protein content by ELISA **(A)**. Microglial cultures were also exposed to different doses of db cAMP (0 to 10 mM) **(B)** as shown and culture supernatants were analyzed for IGF1 by ELISA. Microglial culture IGF1 levels were low (at the lower detection range of the ELISA) and they were further decreased by proinflammatory stimulants. Mean ± SD, One-way ANOVA with Dunnett’s multiple comparisons * *P* <0.05, ** *P* <0.01, *** *P* <0.001.

We next performed ELISA for IGF1 production in human microglia. Microglial cultures were treated with various cell stimuli as shown for Q-PCR analysis, and the 24 h cultures supernatants were subjected to ELISA assay (R&D DuoSet, sensitivity ≈50 pg/ml). As shown in Figure 4A, the amounts of IGF1 protein in microglial supernatants were relatively low (~100 pg/ml range) and the samples stimulated with proinflammatory mediators showed even lower levels. Microglial IGF1 levels were suppressed by IFNγ or LPS, but IL-4 had no effect. The IFNγ effect was dose-dependent, with suppressive effects shown only in high concentrations (100 ng/ml or above). In addition to inflammatory mediators, we tested additional compounds that have been shown to modulate IGF1 expression in other cell types [40,41]. Of these, the cAMP analog db cAMP (0.5 to 5 mM) increased microglial IGF1 production (Figure 4B). However, growth hormone (1 to 100 ng/ml), the primary inducer of hepatic IGF1, had no effect (data not shown). These results together show that IGF1 expression is cell-specific and that human microglial IGF1 production is suppressed by proinflammatory mediators (IFNγ or LPS), is not significantly modulated by IL-4, and is increased by cAMP (see Discussion).

### Regulation of microglial IGF2 protein expression

**Figure 5:**
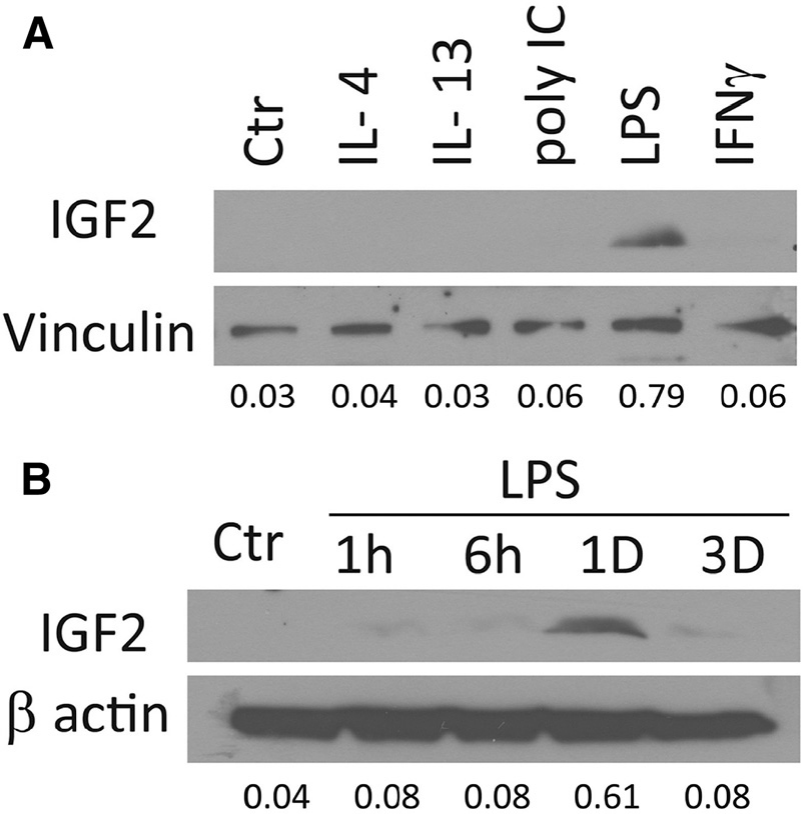
**IGF2 protein expression in human microglia is upregulated by LPS.** Microglia cultures were stimulated with different stimuli, as described for Q-PCR assay, and Western blot analysis was performed for IGF2 protein in a 4 to 20% polyacrylamide gradient gel using a commercial antibody. Densitometric ratios are shown. Induction of IGF2 (≈7.5 kDa) by LPS is shown in microglial samples treated for 24 h **(A,B)**. Results are representative of three independent experiments.

We next attempted to determine the IGF2 protein levels in microglial cultures by ELISA. A commercially available ELISA that is compatible with culture supernatants was obtained from Antigenix America but this had a detection limit of ~300 pg/ml. Other commercial IGF2 ELISAs (Alpco Diagnostics or Diagnostic System Laboratories, for example) were not intended for tissue culture fluid assays and had a similar low sensitivity. Microglial culture IGF2 levels were not detectable using these assays (not shown), perhaps because of the assays’ low sensitivity. Therefore, we next determined microglial IGF2 expression using Western blot analysis (Figure 5). Cell lysates were separated in a 4 to 20% TGX polyacrylamide gradient gel and the blot was probed with a rabbit anti-IGF2 antibody (Abcam), as described. Representative results with densitometric ratios are shown in Figure 5. A single band of ≈7.5 kDa (consistent with the molecular mass of IGF2) was detected in microglial cultures stimulated with LPS but not with IL-4, IL-13, IFNγ, or poly(I:C) (Figure 5A). Time course analyses showed that IGF2 upregulation by LPS was transient at 24 h (Figure 5B). These results together demonstrate that IGF2 expression in human microglia is upregulated by LPS.

### Expression of IGF1 receptor (IGF1R) in mixed neuronal glial cultures

**Figure 6:**
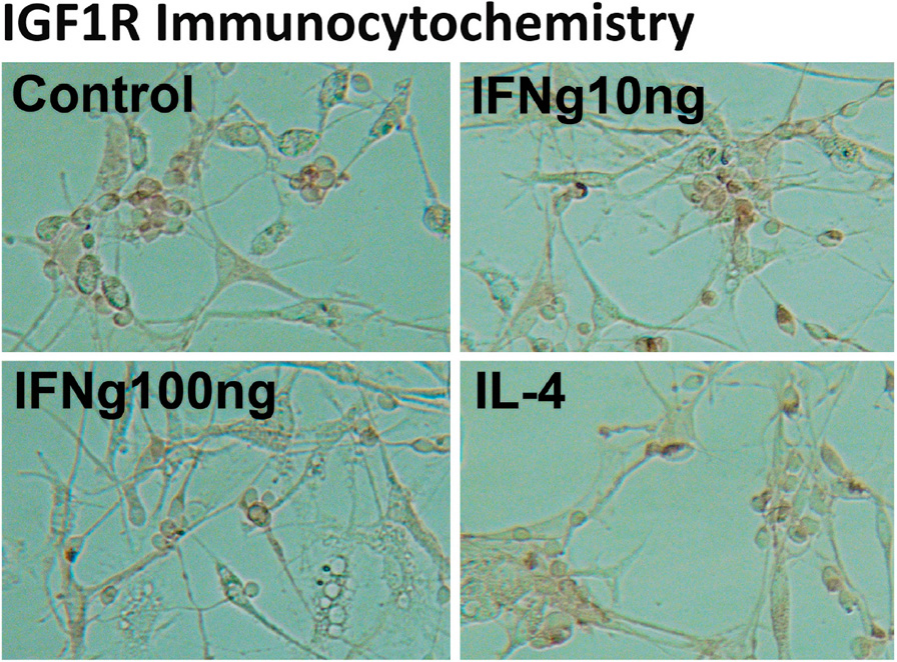
**IGF1R expression in human fetal neurons.** Mixed neuronal glial cultures stimulated for 24 h with IFNγ (10 or 100 ng/ml), IL-4 (10 ng/ml), or LPS (not shown) or not (Control) and immunostained for IGF1R using a commercial antibody. IGF1R immunoreactivity (brown) was selectively expressed in small process-bearing cells in these cultures (neurons) without appreciable differences in the expression levels among various culture conditions.

Both IGF1 and IGF2 signal through the IGF1R, suggesting that both IGFs could promote neuronal survival by activating the downstream Akt pathway, but a direct demonstration of IGF peptides’ neurotrophic effects is rare. We thus examined whether IGF1 and IGF2 confer neuroprotection in our well-characterized cytokine-induced neuronal death assay using primary human neuronal glial cultures [34,37]. First, we examined IGF1R expression by immunocytochemistry of mixed human fetal CNS cultures using an antibody that detects the extracellular domain of human IGF1R (R&D Systems). As shown in Figure 6, IGF1R expression was found predominantly in neurons (small process-bearing cells) and the immunoreactivity was localized to the cell membrane. Glial cells did not appear to have significant IGF1R immunoreactivity. Stimulation of the mixed cultures with various inflammatory mediators did not affect the amount of IGF1R immunoreactivity appreciably (Figure 6).

### IGF1 and IGF2 rescue human fetal neurons from cytokine-induced death

**Figure 7:**
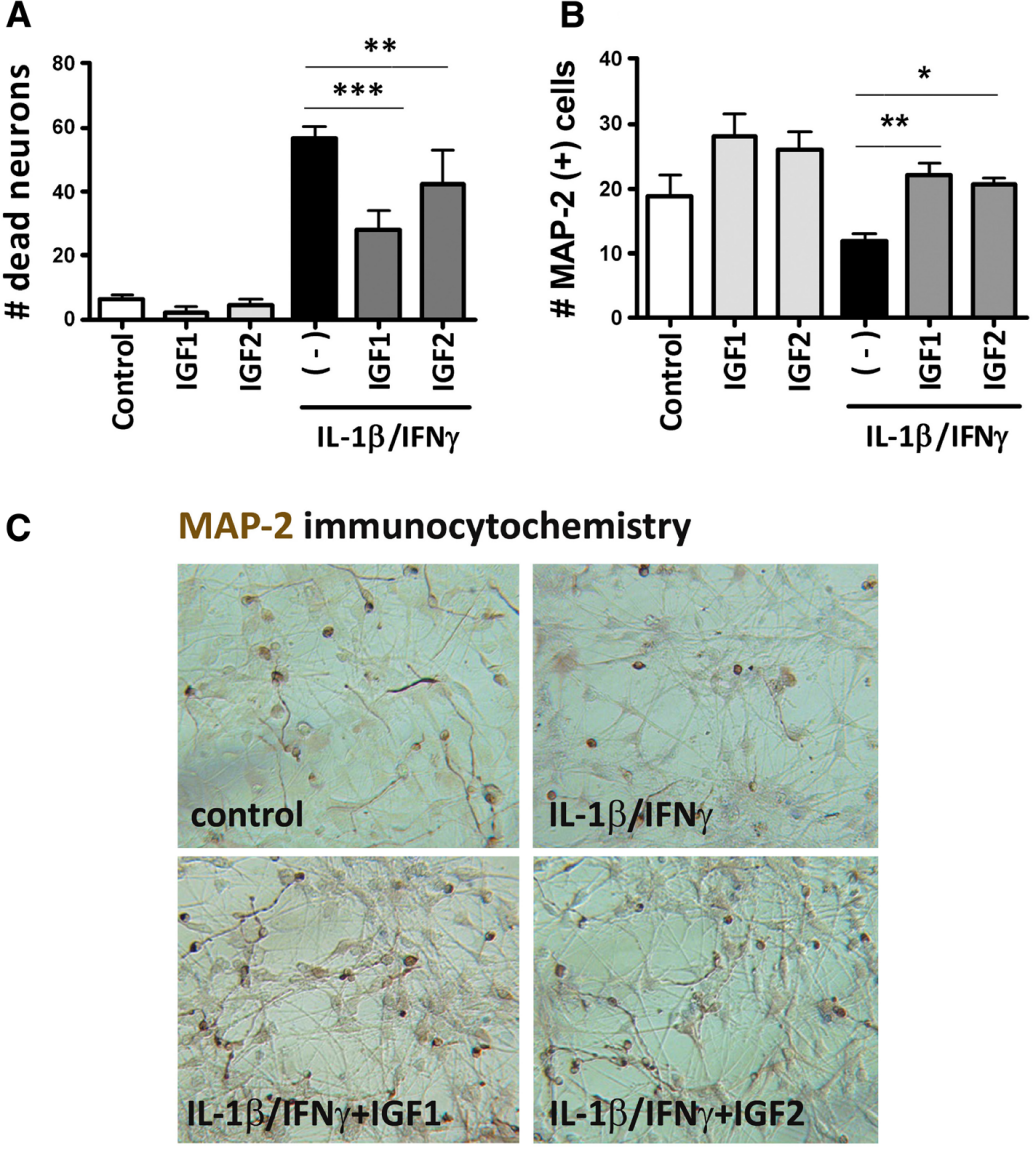
**IGF1 or IGF2 significantly protects neurons from cytokine-induced toxicity.** Mixed human fetal neuronal glial cultures were stimulated with IL-1/IFNγ for 3 days to induce neurotoxicity. A subset of cultures was pre-treated with either IGF1 or IGF2 at 10 ng/ml. Neuronal death was evaluated using viral dye exclusion **(A)** or MAP2 immunostain **(B,C)** as previously described [37]. IGF1 or IGF2 reversed the neuronal dead caused by IL-1/IFNγ Results are mean ± SD from triplicate cultures, and are representative of five independent experiments with similar results. One-way ANOVA with Dunnett’s multiple comparison tests. *P < 0.05, ** *P* < 0.01, *** *P* < 0.001.

We next investigated whether IGFs have the neuroprotective effects in our mixed human fetal CNS cultures, following a published protocol [34,37]. Briefly, cultures consisting primarily of neurons and astrocytes and a minor population of microglia were stimulated with IL-1β/IFNγ (10 ng/ml each) for 3 days to determine the extent of neuronal death. We examined the role of IGFs by treating the cultures with either IGF1 or IGF2 at 10 ng/ml for 2 h prior to cytokine stimulation. Three days later, neuronal death was determined by vital dye exclusion (Figure 7A) or MAP2 immunostain (Figure 7B and C). The results show that IL-1β/IFNγ-induced neuronal death was significantly reduced by addition of recombinant IGF1 or IGF2. Higher concentrations of IGFs (100 ng/ml) or the combination of IGF1 and IGF2 did not confer further protection (data not shown).

## Discussion

Our study demonstrates that microglia and macrophages are the main expressors of IGF1 in human CNS parenchyma. In addition, a number of other cell types located in the subarachnoid space, the cerebrospinal fluid (CSF) brain barrier, and the blood brain barrier (such as meninges, ependymal cells, endothelial cells, and vascular smooth muscle cells) expressed IGF1 immunoreactivity. These results are consistent with the previous studies of developing rodents and human brains, which showed that IGF1 mRNA is predominantly expressed by CNS mesenchymal cells (meninges, enclosed vessels, macrophages, choroid plexus, and so on) [42-44]. Additional recent rodent studies of experimental CNS conditions reported that microglia (and astrocytes in some instances) express IGF1 [45-48]. Less information is available on IGF2, but there are reports that show that microglia in developing and injured CNS express IGF2 protein [49-51]. In addition, intracranial mesenchymal cells serve as sources of IGF2 expression, as for IGF1 [42-44]. Our attempts to delineate IGF2 expression in human CNS have resulted in minimal staining of brain parenchyma. There are several possibilities for this, including extremely low levels of expression and inadequate antibodies for tissue staining. Additional possibilities include rapid capture and degradation of IGF2 by the receptor IGF2R, which we found to be significantly upregulated in activated microglial cells in the CNS [29].

More importantly, we show that cultured human microglia are significant sources of IGF1 and IGF2. Mouse bone marrow macrophages and microglia have been reported to produce ng/ml levels of IGF1 [6,52,53]. Furthermore, murine macrophage and microglia IGF1 production is upregulated by IL-4 or IL-13 [52], but downregulated by IFNγ [6,53], suggesting that IGF1 production is a function of M2 activation. Yet another study of rat microglia showed that IGF1 (and IGF2) are induced by LPS [50]. In the current study, we find that human fetal microglia produce relatively low levels (~100 pg/ml) of IGF1 and that this level is potently suppressed by LPS (and poly(I:C)), as well as by very high concentrations of IFNγ. The expression profile of TNFα mRNA in microglial culture (Figure 2D) suggests that TNFα might mediate the IGF1-suppressive effects of LPS, poly(I:C), and even IFNγ in these cultures, establishing an interesting relationship between IGF1 and TNFα.

Indeed, there is evidence that proinflammatory stimuli suppress neuronal growth factor production. We have recently reported that LPS potently suppresses another microglial-derived neuronal growth factor, progranulin [11]. It has also been shown that BDNF is suppressed by LPS in the rat CNS *in vivo*[54]. These results together establish a relationship between macrophage and microglia activation phenotypes and IGF1 production, and further suggest that neurotrophic growth factor production is suppressed in a proinflammatory (M1) environment but is encouraged in an M2 environment. In addition to macrophage cell types, proinflammatory cytokines have also been shown to inhibit IGF1 expression in systemic cells. For instance, IL-1 and TNFα suppress (growth hormone-induced) IGF1 mRNA expression in hepatocytes [55,56].

Despite extensive study, the molecular mechanism underlying regulation of IGF1 transcription is not well understood. The 5^′^ regulatory region of the IGF-1 gene contains a cAMP response element (CRE) [57,58]. Human microglial IGF1 expression was increased by db cAMP, suggesting that IGF1 expression in these cells is also under the control of CRE. As many neurotransmitters (norepinephrine, dopamine, and so on) activate the cAMP/protein kinase A (PKA) pathway, these results suggest that IGF1 expression can be facilitated in the neural environment. Conversely, under chronic neurodegenerative conditions (such as Alzheimer’s disease and Parkinson’s disease), growth factors levels can be depleted, as a result of selective neurotransmitter deficiencies. The effect of proinflammatory mediators on IGF1 expression might in part be explained as a consequence of their role in cAMP/CRE activation. Together, these results indicate intriguing relationships between neural environment, growth factor production, and neuroinflammation [59,60].

Regulation of IGF2 has been extensively studied in tumors, with little information available on non-neoplastic cells. IGF2 is an imprinted gene and has an important role during mouse development [16,20,25,61]. Excess IGF2 is produced during fetal stage and failure of removal of excess IGF2 due to IGF2R deficiency results in fatal organ overgrowth due to overstimulation of IGF1R. Less is known about the post-developmental role of IGF2, but in human beings, IGF2 production continues after birth. IGF2, similar to IGF1, signals through IGF1R with resulting cell survival, growth, and metabolic effects mediated by the PI3K/Akt pathways. In our investigation, IGF2 expression was uniquely upregulated by LPS (in contrast to IGF1) in human microglia, (and by IL-1β in astrocytes in culture), suggesting that the two IGF peptides are under different regulatory controls. Several sequences identified in the human IGF2 promoter include Sp1, Egr-1-like, C/EBP, and AP-1 sites [61], some of which are inducible by LPS. Future studies are necessary to elucidate the molecular mechanisms underlying differential microglial IGF peptide expression.

IGF1 is a well-known survival factor for many different cell types, including neurons. There is evidence that decreased IGF1 levels are correlated with impaired cognitive function and neurodegeneration in humans. For instance, increased serum TNFα and decreased IGF1 levels were reported in Alzheimer’s patients, with the two showing a significant negative correlation [26]. In another study, CSF (but not serum) levels of IGF1 were found to be diminished in individuals with motor neuron disease [62]. Studies that directly investigated the neurotrophic effects of IGF1 are rare. IGF1, in combination with erythropoietin, has been shown to mitigate HIV gp120-induced neurotoxicity in mouse in both *in vivo* and *in vitro* models [63]. IGF1 was neuroprotective against TNFα-induced toxicity or HIV-infected cell supernatants-induced toxicity in rodent cell lines [64]. Recent studies have assessed the efficacy of exogenous IGF1 in reducing the inflammatory response of rat astrocytes [65]. In contrast to IGF1, little is known about the physiological function of IGF2 in the nervous system. In the mouse, IGF2 has recently been proposed as a critical component in memory enhancement and consolidation via promoting survival and maturation of hippocampal neurons [66,67]. In this study, using human cells, we show that IGF1R is predominantly expressed by neurons, and that recombinant IGF1 or IGF2 individually confer significant protection from cytokine-induced neuronal death and provide sustenance of MAP2 protein expression. These results together support the notion that IGF family peptides are significant survival factors for human or rodent neurons and that IGF1 and IGF2 might provide future therapeutic targets for neurodegenerative diseases.

## Conclusions

In this study, we investigated whether IGF1 and IGF2 are expressed in human microglia *in vivo* and *in vitro*, and whether their expression is modulated by inflammatory cytokines. We also investigated whether IGF1 or IGF2 modulate neuronal survival. We demonstrated that IGFs are expressed in microglia and that their expression is modulated differently; for example, LPS potently suppressed IGF1 but increased IGF2. Lastly, we found that both IGF1 and IGF2 conferred strong protection against cytokine-mediated neuronal death in human fetal neuronal culture, supporting their potential future therapeutic applications in various human CNS conditions. Our results suggest that neuronal growth factors (IGF1, progranulin) are negative regulated by proinflammatory stimuli and that the negative impact of inflammation on neural growth factor production might contribute to neurodegeneration.

## Abbreviations

ANOVA: analysis of variance; AP-1: activation protein-1; BDNF: brain-derived growth factor; BSA: bovine serum albumin; C/EBP: CCAAT/enhancer binding protein; CNS: central nervous system; CRE: cAMP response element; CSF: cerebrospinal fluid; DMEM: Dulbecco’s modified Eagle’s medium; ELISA: enzyme-linked immunosorbent assay; FBS: fetal bovine serum; GH: growth hormone; H&E: hematoxylin and eosin; HIVE: HIV encephalitis; IFNγ: interferon-gamma; IGF1: insulin-like growth factor 1; IGF2: insulin-like growth factor 2; IGF1R: insulin-like growth factor 1 receptor; IGF2R: insulin-like growth factor 2 receptor; IGFBP: insulin-like binding protein; IL-1β: interleukin-1 beta; iNOS: inducible nitric oxide synthase; LPS: lipopolysaccharide; M1: macrophage classical activation phenotype; M2: macrophage alternative activation phenotype; MGC: multinucleated giant cell; NGS: normal goat serum; PBDA: porphobilinogen deaminase; PBS: phosphate-buffered saline; PKA: protein kinase A; Q-PCR: real-time reverse-transcription PCR; TLR: toll-like receptor; TNF: tumor necrosis factor

## Competing interests

The authors declare no competing interests.

## Authors’ contributions

HS, MZ, LD, and NC performed the experiments and interpreted the data; HS and SCL designed the experiments, analyzed the data, and wrote the paper. All authors have read and approved the final version of the manuscript.
